# The methylation pattern of osimertinib resistance: prospects for diagnosis and treatment of NSCLC

**DOI:** 10.3389/fonc.2026.1943703

**Published:** 2026-09-22

**Authors:** Yiting He, Chunxiao Shen, Dan Cheng, Lushun Zhang, Ting Li, Ziying Liu, Xianxin Liu, Liangliang Wang, Xiaorong Zhang

**Affiliations:** 1 Department of Pathology, Clinical Medical School of Jiujiang University, Jiujiang, Jiangxi, China; 2 Jiujiang Clinical Precision Medicine Research Center, Jiujiang, China

**Keywords:** DNA methylation, epigenetics, hypermethylation, hypomethylation, lung cancer, RNA methylation

## Abstract

Osimertinib, a third-generation EGFR-TKI, is effective for non-small cell lung cancer (NSCLC), but drug resistance significantly worsens patient outcomes. Epigenetic regulation, particularly methylation, is crucial in tumor progression and resistance development. Changes in polygenic methylation patterns are linked to osimertinib resistance in NSCLC, affecting drug target mutations, transporter functions, and signaling pathways. However, the mechanisms by which epigenetic regulation impacts NSCLC and drug resistance are not fully understood. This review summarizes the role of polygenic methylation in osimertinib resistance in NSCLC, examining its effects on drug targets, transporter functions, and signaling pathways. It systematically analyzes recent literature on how changes in DNA and RNA methylation patterns affect EGFR mutation status, drug transporter function, and intracellular signaling, providing a theoretical framework for understanding osimertinib resistance. Further research on the interaction between DNA methylation and other resistance mechanisms may lead to new treatment strategies and drug targets, enhancing efficacy and patient survival rates in NSCLC.

## 1 Introduction

Lung cancer, primarily non-small cell lung cancer (NSCLC), remains a leading cause of cancer-related deaths globally (1). NSCLC development is frequently driven by environmental factors, smoking, and aging, with a substantial proportion of patients harboring EGFR mutations that correlate with poor clinical outcomes (2). Osimertinib, a third-generation EGFR-TKI, is effective for treating EGFR mutation-positive NSCLC, improving survival and reducing brain metastases risk (3). However, resistance to osimertinib, due to complex mechanisms like epigenetic changes, remains a challenge (4). Despite the prevalence of NSCLC, research on osimertinib resistance is limited, leaving a significant knowledge gap (5).

DNA methylation, an epigenetic modification catalyzed by DNA methyltransferases (DNMTs), modulates gene expression by adding a methyl group to cytosine residues without altering the underlying DNA sequence (6). This reversible process, involving *de novo* or maintenance pathways, can be undone by TET enzymes (**Figure 1**). In non-small cell lung cancer (NSCLC), DNA methylation disrupts gene expression, promoting tumor growth and drug resistance by silencing tumor-suppressor genes and activating oncogenes. Hypermethylation of specific genes is linked to chemoresistance in NSCLC. For example, the REMAIN system, highlighted in Molecular Cancer, enables targeted therapy by reversing methylation-induced silencing of miR-126-3p, restoring its tumor-suppressive function (7).

**Figure 1:**
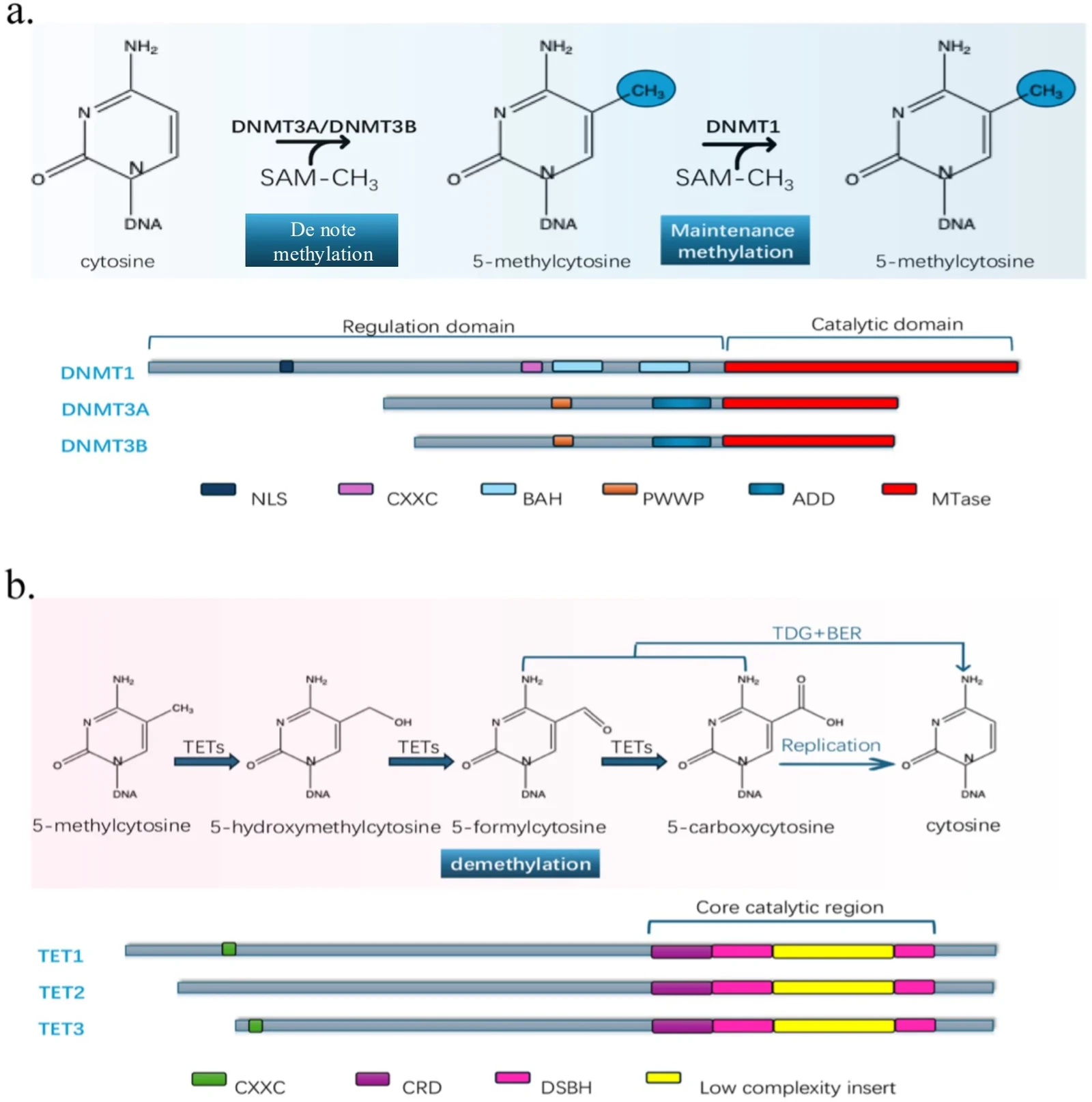
Mechanisms of DNA methylation and demethylation. **(a)** The methylation process involves the transfer of a methyl group from S-adenosylmethionine (SAM), a universal methyl donor, to the 5-carbon position of the cytosine ring in CpG dinucleotides, yielding 5-methylcytosine (m5C). **(b)** Active demethylation involves the oxidation of m5C to other forms of modified cytosines, which can then be removed or replaced.

N6-methyladenosine (m6A) is the main RNA methylation type, crucial for biological functions, involving m6A methyltransferases (writers), demethylases (erasers), and binding proteins (readers) (8). The methyltransferase complex includes METTL3, METTL14, WTAP, and KIAA1429, while FTO and ALKBH5 are key demethylases (9). Binding proteins include YTH-domain proteins, IGF2BPs, and SND1, all of which affect RNA metabolism, mRNA stability, and translation. Abnormal expression of proteins like METTL3, METTL14, FTO, ALKBH5, and YTHDF1–3 is linked to cancer progression and treatment resistance (10–12). For instance, silencing KIAA1429 increases LUAD cell sensitivity to gefitinib, and YTHDF1 is studied for its role in immune-checkpoint inhibitor resistance. m6A demethylation is also linked to chemotherapy resistance, EMT in breast cancer, and metastasis promotion (13, 14).

Multi-gene methylation is closely linked to osimertinib resistance in EGFR-mutated lung adenocarcinoma. In patients who have developed resistance to osimertinib, liquid biopsies of plasma cell-free DNA frequently reveal secondary resistance alterations and associated signaling modifications (15). Furthermore, genome-wide and epigenetic profiling of osimertinib-resistant NSCLC models reveals specific DNA methylation changes that correlate with epithelial-mesenchymal transition (EMT) and therapeutic failure. Distinct from broad-spectrum chemotherapy resistance, epigenetic remodeling in this context directly modulates third-generation EGFR-TKI treatment outcomes. This review specifically synthesizes current evidence regarding polygenic DNA and RNA methylation patterns directly associated with osimertinib resistance in NSCLC, aiming to provide a robust theoretical framework for clinical diagnosis and targeted intervention (16).

## 2 Basic overview of the involvement of DNA methylation in osimertinib resistance

### 2.1 Changes in the DNA methylation status of the mutated *EGFR* gene and related genes

EGFR gene expression is tightly controlled by promoter methylation, affecting transcription and cell function. Changes in DNA methylation, alongside mutations like C797S, are crucial in developing osimertinib resistance in NSCLC patients. These methylation alterations can occur in the EGFR gene or its regulatory network, reshaping gene expression and increasing tumor resistance to treatment (17) (**Table 1**).

**Table 1:** EGFR-associated gene methylation profiles.

Methylation changes in tumors	Gene	Pathways	Reference	Publication year
Methylation	AXL	Gas6-Axl	(18)	2024
	BVES	Cell shape and movement	(19)	2024
	Caspase-8	Cell adhesion、 apoptosis	(20)	2024
	CDO1	Cellular metabolism	(21)	2024
	ESRP1	Cell Aggregation、 Signaling	(22)	2024
	HOXA7	Cell proliferation、 mRNA and protein expression、PI3K/Akt	(23)	2024
	HOXB4	signaling pathway	(24)	2024
	HOXD8	Signaling	(25)	2024
	IGF2BP1	Transcriptional regulation	(26)	2024
	PTGER4	cell proliferation	(24)	2024
	P53	Signaling	(27)	2024
	PTEN	Cell cycle and differentiation	(28)	2024
	RARB	PI3K/AKT signaling pathway	(29)	2024
	RUNX	Cell、proliferation、apoptosis	(30)	2024
	RB	Apoptosis	(31)	2024
	SOX17	Cell cycle regulation、 DNA repair	(21)	2024
	SORT2	immunity	(24)	2024
	BRCA1	NF-kB signaling pathway	(24)	2023
	B3GNTL1	DNA damage repair、EMT	(24)	2023
	CDKN2A(p16)	Signaling、 Epigenetic Regulation	(32)	2023
	DAPK1	DNA damage repair	(33)	2023
	HOXA9	PI3K/AKT	(34)	2023
	STK11	Signaling	(34)	2023
	SMARCA4	cell proliferation、metabolism and metastasis	(32)	2023
	ZNF808	DNA damage repair	(24)	2023
	FHIT	Transcriptional regulation	(35)	2022
	SOCS-7	cell proliferation、metabolism and metastasis	(35)	2022
	CDH1	immunity	(36)	2021
	DLC1	Calcium-dependent cell adhesion	(37)	2021
	KEAP1	DNA repair、Cell cycle regulation	(27)	2021
	SOCS-3	DNA damage repair	(38)	2021
	CDH13	JAK/STAT signaling pathway	(39)	2020
	PRPH	Cell adhesion	(40)	2020
	RASSF1A	Cell Cytoskeleton	(41)	2020
	SPINT1/2	Apoptosis	(42)	2020
	TAC1	Transcriptional regulation、cancer development and progression	(21)	2020
	ING1	Signaling	(31)	2019
	ITGA4	cell cycle regulation、 DNA repair Cell migration、and cell adhesion	(24)	2019
Demethylation	HAI-1	Signaling	(43)	2024

### 2.2 The role of DNA methylation in osimertinib resistance

Emerging evidence indicates that specific DNA methylation patterns, especially in gene promoters, can predict responses to osimertinib and contribute to resistance (44). Tumor-suppressor gene promoters in cancer cells often exhibit abnormal hypermethylation due to DNA methyltransferases (45). Methylation is crucial for cancer growth, particularly in PRC2-deficient cancers. Studies show that DNA hypomethylation can inhibit cancer by activating tumor-suppressor genes, suppressing oncogenes, and inducing a “viral mimicry” state. This response can enhance therapy sensitivity or trigger apoptosis, highlighting DNA hypomethylation as a promising target for anti-cancer therapy (46).

#### 2.2.1 DNA methylation contributes to osimertinib resistance by affecting the expression of related genes

The EGFR C797S mutation, a common resistance mutation in NSCLC patients treated with osimertinib, occurs in exon 20 of the EGFR gene and disrupts drug binding, leading to resistance (47). Its frequency, along with L718V and L718Q mutations, varies by EGFR mutation type (48). Rather than directly promoting this genetic mutation, it is hypothesized that DNA methylation can indirectly contribute to osimertinib resistance by affecting EGFR gene expression and altering EGFR signaling or related pathways like PI3K-AKT (**Figure 2**). Similarly, while MET amplification is a distinct copy-number variation, DNA methylation can also upregulate the MET receptor tyrosine kinase signaling by influencing its transcription or related genes. EMT can change signaling pathways, cell phenotypes, and drug metabolism, affecting LUAD cells’ sensitivity to osimertinib. While MET amplification’s impact on tumor behavior and the use of MET inhibitors to overcome resistance are well-studied, research on the direct link between MET amplification and specific DNA methylation patterns is limited. Genetic alterations like the EGFR C797S mutation and genomic changes like MET amplification are crucial resistance mechanisms in NSCLC, hypothetically connected to epigenetic modifications like DNA methylation, but their clinical importance and mechanisms remain unclear due to insufficient research (49). Future studies should employ high-throughput sequencing and epigenomics to investigate these links, potentially revealing new targets and strategies for precise NSCLC treatment (50, 51).

**Figure 2:**
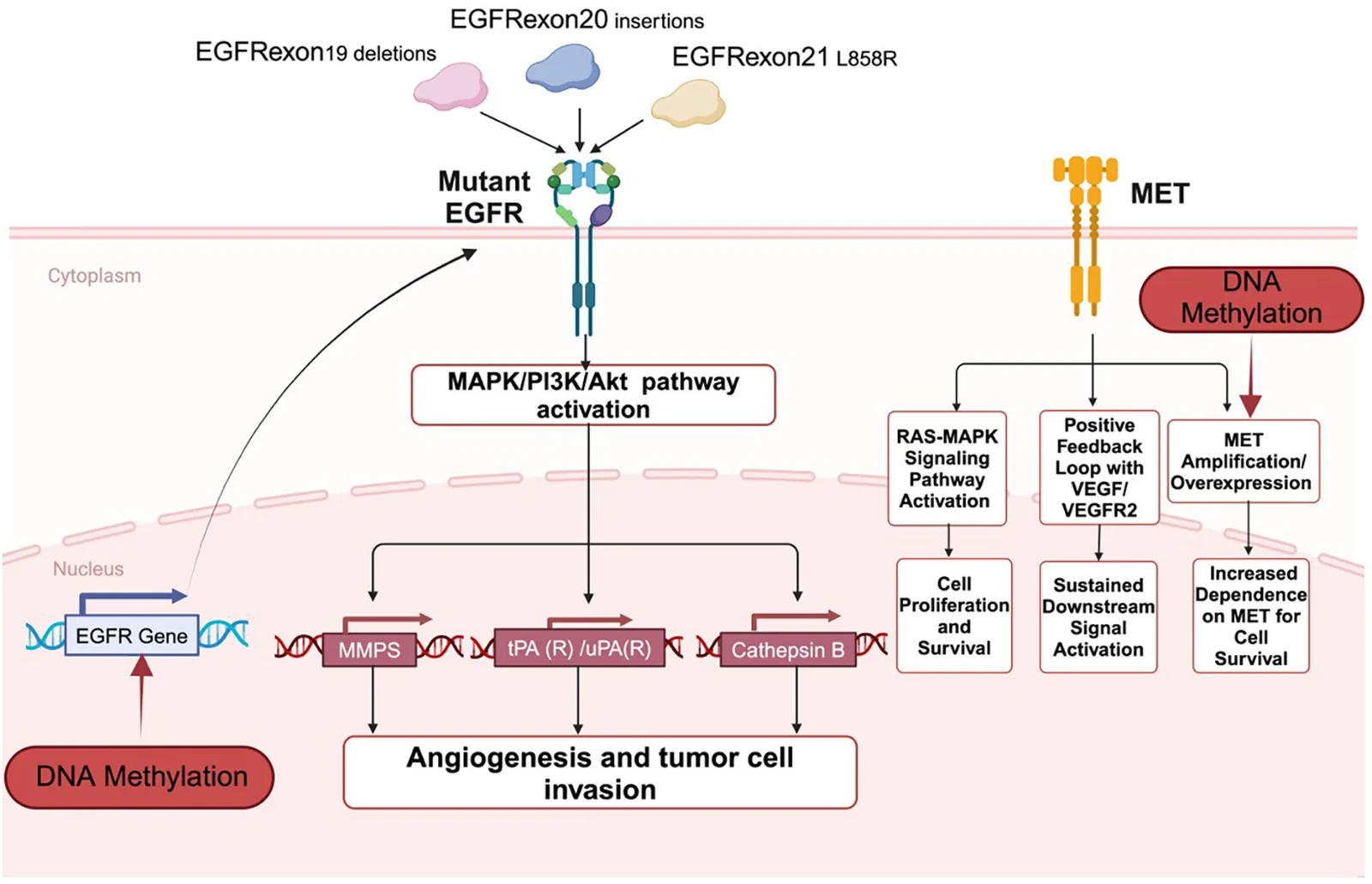
The roles of EGFR and MET in non-small cell lung cancer (NSCLC) biology. EGFR and MET are key drivers of NSCLC. Resistance to EGFR inhibitors, such as osimertinib, is often linked to MET amplification. The combined targeting of EGFR and MET is an emerging strategy for combating resistance to these inhibitors in advanced disease. DNA methylation influences EGFR and MET in NSCLC, potentially mediating resistance to osimertinib and other targeted therapies.

#### 2.2.2 The relationship among upregulated DNA methyltransferase expression, downregulated TET expression, increased DNA methylation, and osimertinib resistance

In osimertinib-resistant cells, increased DNMT1 expression forms a complex with UHRF1, boosting NF-κB activity and contributing to drug and radiotherapy resistance (36) (**Figure 3**). This complex also downregulates MUC17, impacting mucosal barrier maintenance and tumor regulation, which affects cell sensitivity to osimertinib (52). MUC1-C activates DNMT1 and DNMT3B, altering DNA methylation and activating the MET, EGFR, ERK, and AKT pathways. TET2 deficiency triggers NF-κB signaling, leading to EGFR-TKI resistance. In cells with TET2/DNMT3A mutations, the epigenetic agent T-dCyd inhibits DNMT1, reducing cell viability and inducing apoptosis, showing anti-tumor potential by affecting DNA methylation (53). Studies indicate that TET2 expression levels significantly influence LUAD growth and spread. High TET2 expression inhibits LUAD proliferation and metastasis, while silencing it enhances these abilities. TET2 suppresses LUAD initiation and metastasis by activating the cGAS-STING pathway (54).

**Figure 3:**
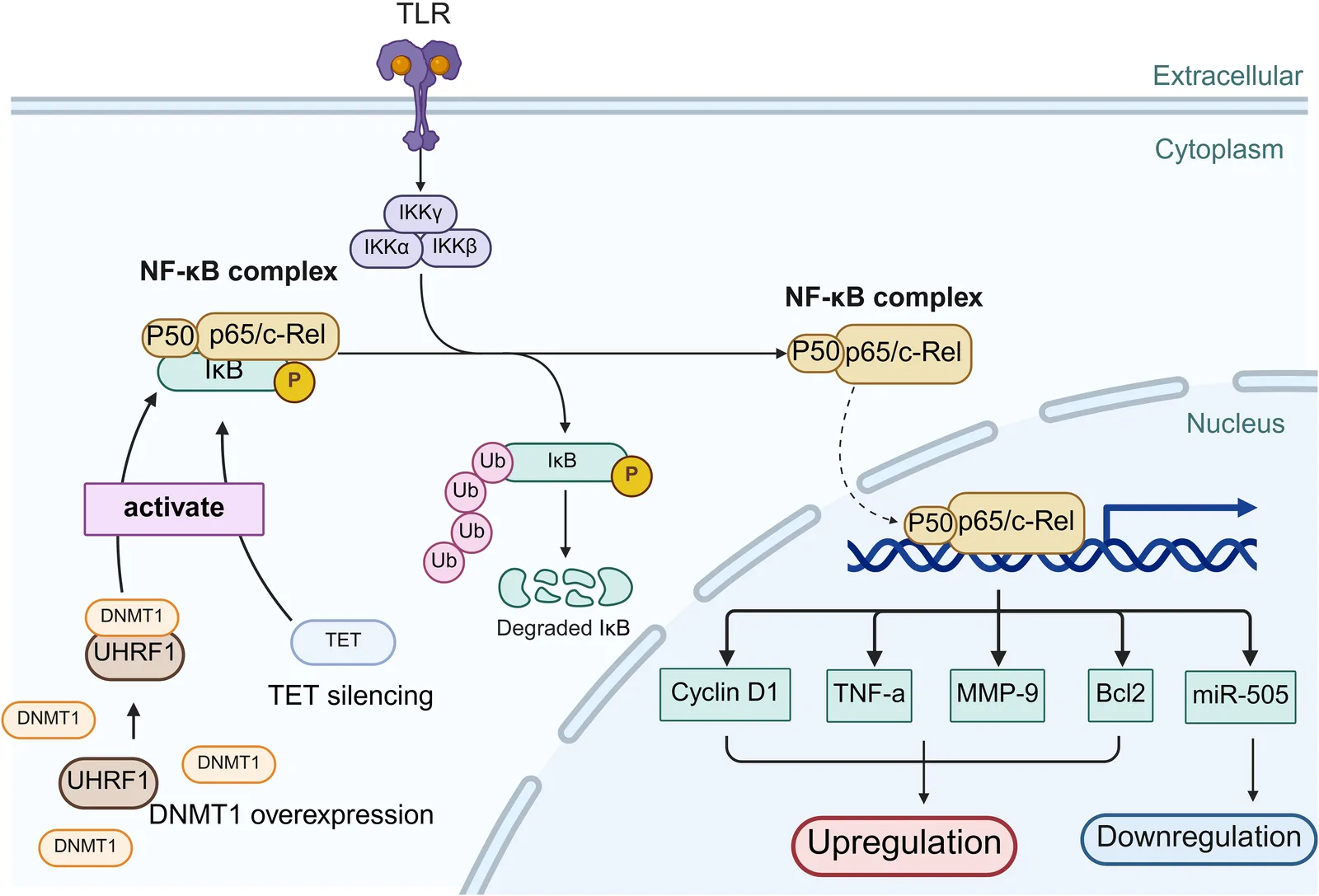
The impact of DNA methylation on osimertinib targets. The overexpression of DNMT1 and the silencing of TET can activate NF-κB, which, in turn, influences metastasis, invasion, and chemoresistance in non-small cell lung cancer by acting on different target genes.

Recently, DNA methyltransferase inhibitors (DNMTis) have emerged as promising cancer treatments by inhibiting DNA methylation to curb tumor growth. Research has improved DNMTis for enhanced DNA hypomethylation with lower toxicity. Studies are investigating DNMTis’ potential to overcome cancer drug resistance, with a notable study showing that combining a DNMT1 inhibitor (5-Aza) with gefitinib or osimertinib restores drug sensitivity in resistant LUAD cells. This underscores the need for more effective, less toxic DNMTis to improve cancer treatment and tackle drug resistance (55).

## 3 Research progress relating to the role of RNA methylation in osimertinib resistance mechanisms

### 3.1 m6A methylation regulates RNA metabolism via multiple mechanisms

#### 3.1.1 The dual regulation of RNA stability and liquid-liquid phase separation

M6A methylation influences RNA stability by either promoting degradation or enhancing stability, depending on the cellular context and RNA-binding proteins (56) (**Figure 4**). This regulation involves liquid-liquid phase separation, creating RNA-protein complexes that determine mRNA fate. In lung cancer, it can advance disease by aiding oncogene expression via RBPs such as FMRP (57).

**Figure 4:**
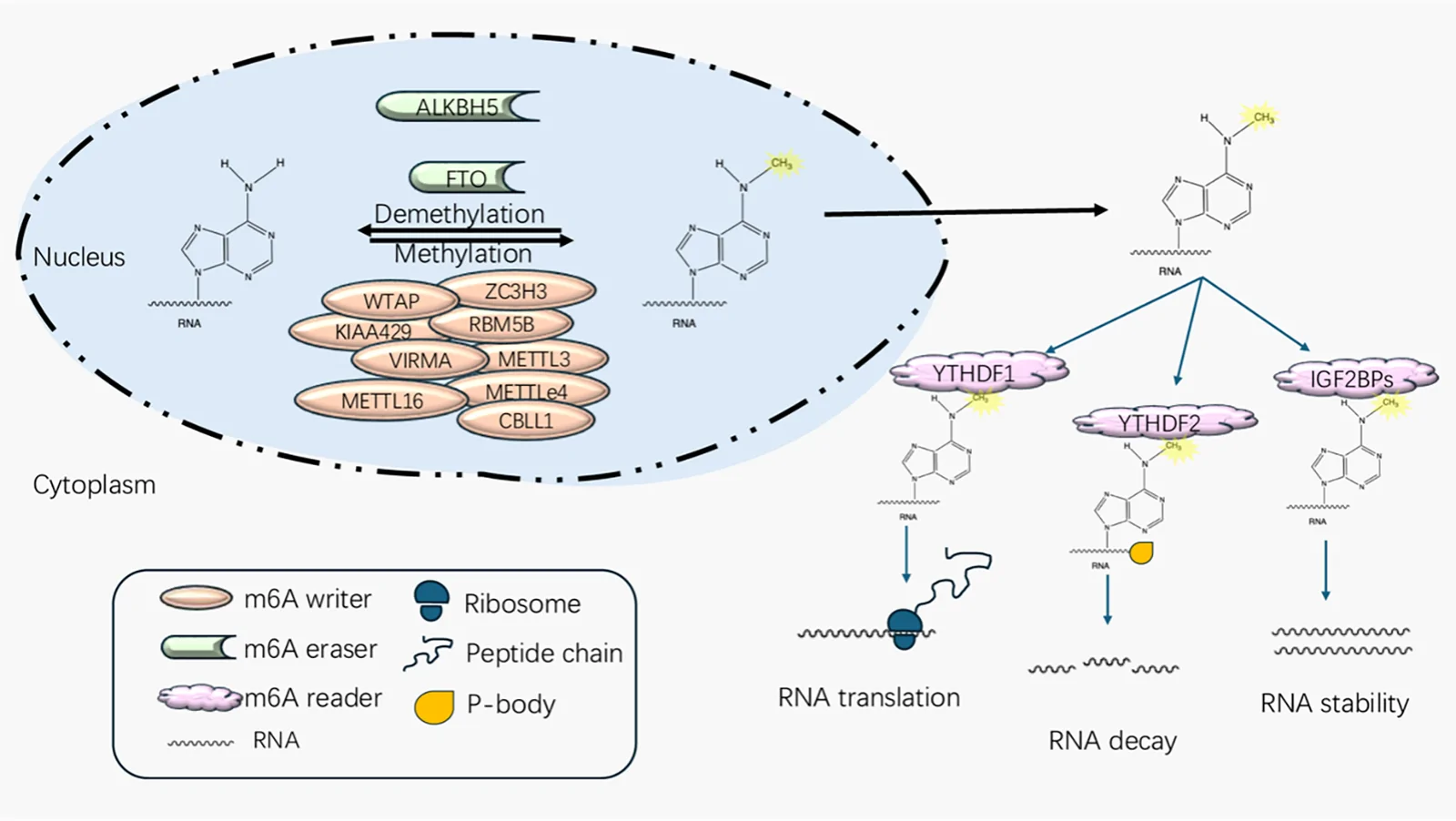
Types and functions of RNA methylation. The effectors of the m6A pathway include m6A writers, m6A erasers, and m6A readers, which exert regulatory functions in the stabilization of genetic material within their respective domains.

#### 3.1.2 The impact on RNA processing: alternative splicing and miRNA biogenesis

M6A methylation is crucial for RNA splicing, where methyltransferases near splice sites aid splicing factor binding, influencing mRNA patterns (58). Demethylases remove m6A, altering splicing and increasing gene expression diversity. M6A also supports miRNA biogenesis, with METTL3/14 and DGCR8 speeding up pri-miRNA maturation (59). Recent research reveals that m6A modifications in 7SK snRNA interact with RNA polymerase II and regulatory factors, preventing promoter pausing and enhancing transcription (60).

#### 3.1.3 The regulation of RNA translation: initiation and efficiency

M6A-modified mRNA is recognized by RNA-binding proteins that attract translation initiation factors, accelerating translation (61). This modification affects translation efficiency via competitive binding. For example, the m6A reader FMR1 binds to m6A-modified mRNA to slow translation by obstructing ribosome movement, whereas YTHDF1 boosts translation (62). This mechanism enables cells to adjust translation rates according to different conditions (63).

#### 3.1.4 The association with other methods of gene expression regulation

M6A and histone modifications create a complex regulatory network, where m6A serves as a crucial node to adjust histone modifications, such as KMT5C, for precise gene expression control, underscoring RNA methylation’s role in this process (64).

### 3.2 The association between osimertinib resistance and RNA methylation

#### 3.2.1 The relationship between RNA methylation and the stability and translation efficiency of EGFR-TKI target mRNAs

Alterations in m6A modification affect mRNA stability and EGFR protein levels, influencing the effectiveness of EGFR-TKIs in the EGFR signaling pathway (65). RNA methylation also affects mRNA translation efficiency, impacting osimertinib’s efficacy (66). YTHDC2, crucial for m6A methylation, binds to lncRNAs like ZNRD1-AS1 to regulate their stability and function (67). A pan-cancer analysis using The Cancer Genome Atlas data revealed that YTHDC2 overexpression significantly stabilizes ZNRD1-AS1, highlighting RNA methylation’s role in lncRNA stability and its potential impact on tumor drug resistance (67).

#### 3.2.2 RNA methylation-related factors that affect the stability and translation efficiency of EGFR-TKI target mRNAs

Research shows that combining TKIs with drugs like chidamide, β-elemene, and meclofenamic acid, or using m6A inhibitors, can decrease drug resistance and inhibit lung cancer cell growth (77). Additionally, evidence suggests that osimertinib-resistant lung cancer patients experience specific RNA methylation changes, affecting both EGFR-TKI target mRNA methylation and the expression of other drug resistance-related genes (78).

RNA methylation contributes to osimertinib resistance by affecting molecules like JUNB, which is vital for cell processes and tumor growth, thus impacting lung cancer progression and EGFR-TKI resistance (79). Additionally, circKRT17 sequesters miRNAs to boost gene expression, counteracting METTL3 knockout effects on cell proliferation and apoptosis (80). The lncRNA LINC01510 promotes MET oncogene transcription. Wnt signaling activation, initiated by ligand-receptor binding, involves β-catenin, GSK3β, and APC, forming a network crucial for growth and disease, with abnormal activation linked to cancer progression and drug resistance (81).

### 3.3 Exploration of the specific mechanisms by which RNA methylation participates in osimertinib resistance in non-small cell lung cancer

#### 3.3.1 m6A modification mediated by m6A writers and erasers participates in osimertinib resistance by regulating mRNA processing and translation

RNA methylation is crucial for osimertinib resistance in NSCLC, affecting tumor cell sensitivity through complex mechanisms. In resistant lung cancer, m6A writers like METTL3 are overexpressed, and their removal inhibits cell growth and triggers apoptosis, highlighting m6A’s role in resistance (82) (**Table 2**). METTL7B is also overexpressed in NSCLC tumors, promoting growth by influencing the cell cycle. ALKBH5, an RNA demethylase, removes m6A modifications to maintain mRNA stability and function. In glioma stem cells, METTL3-mediated m6A modifications destabilize mRNA in the Wnt/β-catenin pathway, leading to overactivation and drug resistance (83).

**Table 2:** The functions of m6A effectors in Osimertinib resistance.

m6A effector	Regulator	Regulation	Target/approach	Reference	Publication year
Writer	METTL3	PR	Wnt/β-catenin、PI3K/AKT、LINC00969 signaling pathways	(68)	2022
	WTAP	PR	ALK/ROS1/c-MET signaling pathway、cell PANoptosis	(69)	2024
	FMR1	PR	Apoptosis、angiogenesis	(62)	2024
	KIAA1429	DR	MAP3K2/JNK/MAPK signaling pathway	(70)	2023
	METTL7B	PR	GPX4, SOD1 and HMOX1 signaling pathways	(71)	2021
Reader	IGF2BPs	PR	COX6B2、PI3K-Akt signaling pathways	(56)	2021
	YTHDC1	PR	Immune evasion	(72)	2023
	YTHDF3	PR	Wnt/Let-7b	(61)	2022
	YTHDC2	PR	ZNRD1-AS1、ASK1/JNK/p38 signaling pathways	(73)	2022
Eraser	ALKBH5	DR	TGFβR2、SMAD3、SMAD6 signaling pathways	(72)	2024
	FTO	DR	ESR1	(74)	2024
Associated Factors	HDGF	PR	PI3K/AKT、MEK/ERK signaling pathways	(75)	2023
	ARNTL2	PR	EMT, ferroptosis	(76)	2022

PR, promote resistance; DR, decrease resistance.

In LUAD cells, METTL3 affects osimertinib sensitivity by regulating circKRT17 through m6A modification (84). High METTL3 levels can lead to treatment resistance and metastasis via the MALAT1-miR-1914-YAP axis and stabilize JUNB mRNA, promoting EMT. METTL3 also stabilizes p53 independently. In EGFR-mutant cells, the p53-R273H mutant induces EMT, increasing resistance to EGFR-TKIs. Conversely, FTO reduces m6A on MZF1 and USP7, promoting lung cancer growth and serving as a prognostic marker (85). A lncRNA from the FTO locus inhibits p53 signaling, suggesting a treatment target. In LUAD, METTL7B stabilizes mRNAs for antioxidant enzymes GPX4, SOD1, and HMOX1 via m6A, helping tumor cells resist osimertinib by reducing reactive oxygen species. Pan-cancer analysis shows METTL7B knockout impairs tumor growth, migration, invasion, and EMT, linking it to NSCLC development (86).

NSUN2, an RNA cytosine C(5)-methyltransferase, modifies methylation in NSCLC by boosting RNA m5C via the NSUN2/ALYREF pathway, aiding Cr(VI)-induced lung cancer. Overexpressing NSUN2 in gefitinib-sensitive cells disrupts m5C modification, stabilizes QSOX1 mRNA through YBX1, and fosters EGFR-TKI resistance. Osimertinib treatment lowers m5C in sensitive cells but raises it in resistant ones. Innate osimertinib resistance links to high NSUN2-mediated m5C, while sensitive NSCLC cells have lower m5C (87).

Western blot analysis shows low ALKBH5 expression in NSCLC, which is associated with cancer growth and spread (88). ALKBH5 inhibits NSCLC progression by reducing YAP expression and activity, partially blocking the TGF-β/SMAD pathway, and affecting EMT through m6A-modified targets. In NSCLC with EGFR mutations, EGFR activation leads to SRC-mediated ALKBH5 phosphorylation, preventing its nuclear export and enhancing mRNA m6A demethylation, possibly contributing to osimertinib resistance and suggesting a new therapeutic target (89).

#### 3.3.2 m6A Readers participate in drug resistance by affecting mRNA translation and RNA stability

Activation of the Wnt signaling pathway decreases Let-7d expression, boosting YTHDF3-mediated m6A modification and promoting circFBXW7-185AA translation (90). This circRNA interacts with β-catenin in an m6A-dependent manner, reducing its stability via ubiquitination and inhibiting the Wnt pathway, potentially leading to osimertinib resistance. A study on primary and metastatic LUAD revealed that IGF2BP2 stabilizes certain RNAs through m6A modification, activates the PI3K-AKT pathway, and promotes angiogenesis and metastasis (91). This pathway can also be activated by ARNTL2, making LUAD cells sensitive to EGFR-TKIs. Similarly, IGF2BP3 enhances target gene translation by promoting the nuclear export and stabilization of m6A-modified mRNAs (92).

#### 3.3.3 Interaction between RNA methylation and other epigenetic modifications

RNA methylation, DNA methylation, and histone modification collectively influence EGFR activation and signaling. KMT5C deficiency elevates LINC01510, causing resistance to EGFR inhibitors in NSCLC through the LINC01510/MET axis. DNA hypermethylation suppresses tumor-suppressor genes like ALKBH5, increasing m6A levels and impacting mRNA processes linked to KRAS and LKB1. This worsens KRAS mutations and LKB1 deletion, contributing to osimertinib resistance in lung cancer (93).

## 4 The value of methylation profiling analysis in the diagnosis of osimertinib resistance

Methylation profiling is crucial for diagnosing Osimertinib resistance (94). A study on NSCLC patients treated with Osimertinib found that methylation levels of certain genes (e.g., RASSF1A, RASSF10) in plasma free DNA and circulating tumor cells (CTCs) were higher at disease progression than at baseline (95). The results showed low consistency between plasma DNA and CTCs, suggesting they provide complementary insights (96). This indicates that DNA methylation profiling may hold promise as a potential diagnostic and predictive biomarker for Osimertinib resistance, though extensive clinical validation involving large, independent cohorts and prospective studies is still warranted (97). Methylation profiling is vital for diagnosing resistance to Osimertinib. A study on NSCLC patients revealed that methylation levels of specific genes, like RASSF1A and RASSF10, in plasma free DNA and circulating tumor cells (CTCs) increased at disease progression. The low consistency between plasma DNA and CTCs suggests they offer complementary insights, indicating that DNA methylation analysis could be a diagnostic tool for Osimertinib resistance (98–100).

## 5 Reversal strategies for osimertinib resistance targeting multi-gene methylation

Targeting epigenetic alterations, particularly aberrant DNA and RNA methylation pathways, has emerged as a promising strategy for overcoming acquired resistance to osimertinib in EGFR-mutated NSCLC.

Abnormal hypermethylation mediated by DNA methyltransferases (DNMTs) frequently silences tumor-suppressor genes and drives drug resistance. Pharmacological intervention using DNA methyltransferase inhibitors (DNMTis) has shown significant potential in restoring drug sensitivity. For instance, combining DNMT inhibitors (such as 5-Aza) with EGFR-TKIs like gefitinib or osimertinib can effectively reverse resistance in resistant lung adenocarcinoma (LUAD) cells. Furthermore, targeting upstream regulatory complexes (such as the DNMT1/UHRF1 complex or MUC1-C-driven methylation pathways) offers novel therapeutic avenues to suppress NF-κB signaling and re-sensitize tumor cells to osimertinib (52).

Aberrant RNA methylation, particularly dysregulated m6A modifications driven by specific writers (e.g., METTL3, METTL7B) and erasers, contributes heavily to osimertinib resistance by stabilizing resistance-associated transcripts or promoting epithelial-mesenchymal transition (EMT). Therapeutic strategies that modulate m6A regulators or utilize combined treatments (such as integrating m6A-related inhibitors with targeted therapy) have demonstrated efficacy in inhibiting resistant cell growth and triggering apoptosis. Disrupting these methylation-dependent cascades provides a robust theoretical foundation for clinical reversal of osimertinib resistance (101).

## 6 Clinical application of multi-gene methylation research results

Research on multi-gene methylation shows promise for clinical use, particularly in cancer diagnosis. Detecting specific gene methylation can aid early diagnosis and prognosis. For instance, in colorectal cancer studies, the methylation status of 57 gene promoter CpG sites was inversely related to gene expression and linked to progression-free survival (102). This information can guide treatment decisions, especially regarding anti-EGFR antibody therapy, despite not being directly related to osimertinib resistance. In lung cancer research, detecting multi-gene methylation in patients resistant to osimertinib aids in understanding resistance mechanisms and guiding clinical treatment. Liquid biopsy analysis of plasma free DNA can reveal gene mutations linked to osimertinib resistance, like MAP2K1 and PIK3CA, allowing for timely treatment adjustments (103). Additionally, methylation profile analysis helps assess patient response to treatments, such as osimertinib combined with voretigene in EGFR mutation-positive and MET amplified NSCLC patients, by evaluating the treatment’s efficacy and safety, thus informing clinical decisions and facilitating the clinical application of multi-gene methylation research (104).

## 7 Conclusion

Methylation is crucial in osimertinib resistance in NSCLC by altering tumor cell sensitivity. DNA methylation, particularly through the action of DNA methylation regulators and methylation-associated alterations, modulates key signaling pathways such as NF-κB and PI3K-AKT, thereby fostering osimertinib resistance. RNA methylation, through m6A modifications, affects gene expression and mRNA stability, contributing to resistance. Both DNA and RNA methylation, along with histone modification, play roles in drug resistance. Advances in technology are expected to reveal more about methylation’s role in resistance, leading to personalized treatments and new therapeutic targets as we better understand methylation abnormalities in cancer.

## Glossary

5-Methylcytosine (m5C): A modified cytosine nucleotide formed by the addition of a methyl group to the 5-carbon position, primarily in CpG dinucleotides, critical for DNA methylation
5-Hydroxymethylcytosine (5hmC): An oxidized form of 5-methylcytosine, intermediate in DNA demethylation pathways
Active Demethylation: A process where methyl groups are enzymatically removed from DNA, catalyzed by TET proteins
ALKBH5: An RNA demethylase that removes N6-methyladenosine (m6A) modifications from mRNA, influencing gene expression
DNA Methyltransferases (DNMTs): Enzymes (e.g., DNMT1, DNMT3A/B) that catalyze the addition of methyl groups to DNA, regulating gene transcription
DNA Methylation: An epigenetic modification involving the addition of methyl groups to DNA, typically at CpG islands, affecting gene expression and cellular processes
Drug Resistance: The ability of cancer cells to survive treatment with targeted therapies like osimertinib, often mediated by genetic or epigenetic alterations
EGFR (Epidermal Growth Factor Receptor): A transmembrane tyrosine kinase receptor, mutations in which drive oncogenesis, particularly in NSCLC
EGFR-TKI (Tyrosine Kinase Inhibitor): A class of drugs (e.g., osimertinib) that block EGFR signaling, used to treat EGFR-mutated NSCLC
Epigenetics: The study of heritable changes in gene expression without alterations to the DNA sequence, including methylation and histone modifications
Epithelial-Mesenchymal Transition (EMT): A biological process where epithelial cells lose polarity and gain mesenchymal characteristics, promoting metastasis and drug resistance
FTO: An RNA demethylase that removes m6A modifications, linked to cancer progression and drug resistance
Hypermethylation: An increase in DNA methylation levels, often silencing tumor-suppressor genes
Hypomethylation: A decrease in DNA methylation levels, potentially activating oncogenes or inducing antiviral immune responses
IGF2BPs: RNA-binding proteins that stabilize m6A-modified mRNAs, promoting translation and oncogenesis
Liquid-Liquid Phase Separation (LLPS): A process where biomolecules form membraneless organelles, regulating RNA metabolism and signaling
m6A Modification (N6-Methyladenosine): The most abundant RNA methylation, affecting mRNA stability, splicing, and translation
MET Amplification: Overexpression of the MET receptor tyrosine kinase, a common resistance mechanism to EGFR-TKIs
METTL3: A methyltransferase (m6A “writer”) that catalyzes m6A modifications, critical for mRNA processing
Non-Small Cell Lung Cancer (NSCLC): The most common type of lung cancer, accounting for ~85% of cases, often driven by EGFR mutations
Osimertinib: A third-generation EGFR-TKI used as first-line treatment for EGFR-mutated NSCLC, targeting both activating and resistance mutations (e.g., T790M)
Passive Demethylation: A reduction in DNA methylation during cell division due to insufficient maintenance methylation
PI3K-AKT Pathway: A signaling cascade involved in cell growth, survival, and metabolism, frequently dysregulated in cancer
RNA Methylation: Chemical modifications to RNA (e.g., m6A, m5C), regulating gene expression post-transcriptionally
TET Proteins (TET1, TET2, TET3): Enzymes responsible for DNA demethylation by oxidizing 5-methylcytosine to 5hmC
YTHDF1: An m6A “reader” protein that enhances translation of m6A-modified mRNAs, linked to immune evasion and drug resistance
ZNRD1-AS1: A long non-coding RNA (lncRNA) regulated by m6A methylation, involved in NSCLC progression and drug resistance.
